# Understanding Categorical Learning in Neural Circuits Through the Primary Olfactory Cortex

**DOI:** 10.3389/fncel.2022.920334

**Published:** 2022-06-24

**Authors:** Tor Stensola, Hanne Stensola

**Affiliations:** Dynamical Systems Neuroscience Collaboratory, Faculty of Health and Sports Sciences, University of Agder, Kristiansand, Norway

**Keywords:** olfaction, novelty, modulatory systems, cortical dynamics, categorical learning

## Abstract

Knowing which elements in the environment are associated with various opportunities and dangers is advantageous. A major role of mammalian sensory systems is to provide information about the identity of such elements which can then be used for adaptive action planning by the animal. Identity-tuned sensory representations are categorical, invariant to nuances in the sensory stream and depend on associative learning. Although categorical representations are well documented across several sensory modalities, these tend to situate synaptically far from the sensory organs which reduces experimenter control over input-output transformations. The formation of such representations is a fundamental neural computation that remains poorly understood. Odor representations in the primary olfactory cortex have several characteristics that qualify them as categorical and identity-tuned, situated only two synapses away from the sensory epithelium. The formation of categorical representations is likely critically dependent on—and dynamically controlled by—recurrent circuitry within the primary olfactory cortex itself. Experiments suggest that the concerted activity of several neuromodulatory systems plays a decisive role in shaping categorical learning through complex interactions with recurrent activity and plasticity in primary olfactory cortex circuits. In this perspective we discuss missing pieces of the categorical learning puzzle, and why several features of olfaction make it an attractive model system for this challenge.

## Introduction

In a complex and changing world, sensory signals can be extremely rich in information and therefore challenging for an animal to process rapidly and adaptively. One way for the brain to mitigate this is through abstraction: sensory structure can be categorized by grouping relevant signals into simpler representations, thereby facilitating interpretation of the sensory scene. Sensory signals emanating from unitary phenomena such as objects, conspecifics or contexts tend to covary in unique ways, meaning correlation structures within the sensory scene can be exploited to encode *identity* of external sensory sources. Because physical information arriving at sensory organs does not completely specify the structure of the environment, however, the brain is tasked with *inferring* what that structure is based on previous experience, a process that requires learning and memory. Knowledge about identity is useful not only for scene interpretation, but for assigning and predicting specific action-outcome contingencies that reduce uncertainty about environment dynamics. Identity-tuned representations are categorical in the sense that the neural response remains unchanged under variation along certain axes of the sensory input space. Categorical sensory representations that provide inference about stimulus identity in the presence of variable, incomplete or ambiguous sensory data are well established across a range of sensory and non-sensory cortical areas. Most of these responses are found in higher-order cortices, several steps away from the sensory input organs. This is in part because the sensory feature axes that these representations become invariant toward tend to be specifically encoded in lower-order cortices. It is hard to understand the transformations that take place during the formation of categorical representations when there are multiple intervening synaptic steps between sensory input and the neural response. The rodent olfactory system offers an attractive model system for studying categorical identity-tuned representations and their formation as this functionality is implemented already in the first cortical processing step, two synapses from the sensory organ.

## Odor Processing in Olfactory Cortex

Odor representations in the primary olfactory cortex are less like low-level feature detectors in other primary cortical counterparts, and more like representations found in higher-order cortices where representational structure principally reflects intrinsically relevant encoding axes such as perceptual categories.

### Olfactory Processing Reflects the Discrete and High-Dimensional Nature of the Olfactory Input Space

The rodent olfactory system has evolved to detect and perceptually classify a staggering range of volatile chemical configurations present in the environment. Olfactory sensory neurons (OSNs) in the nasal epithelium each express one of an estimated 1500 distinct genes devoted to the mouse olfactory receptor (OR) complement (Young et al., 2002). There is not a one-to-one correspondence between chemical stimulus features and OR activation. Instead, ORs are broadly tuned so that each OR is activated by a unique range of chemical features, and each of these chemical features activates a unique range of ORs. The complex interactions between chemical features and ORs make it infeasible to deconstruct the exact chemical makeup that caused the activation, for instance whether the stimulus was monomolecular or a mixture of molecules. What is available to the brain are instead combinatorial OSN activation patterns.

One synapse downstream from the olfactory epithelium, activity in the main olfactory bulb (OB) is organized into anatomically segregated glomeruli that are clusters of axons from OSNs that terminate on dendrites of mitral and tufted cells that then project directly to olfactory cortical areas. Each glomerulus receives inputs from a single type of OR-OSNs. This labeled line organization is not preserved one synapse further downstream in the primary olfactory cortex. Multiple cortical areas can be included in the term primary olfactory cortex (O1) on the criterion that they receive direct input from the OB. Here we focus on the anterior portion of O1 (aO1, see Box 1 for details). aO1 cells receive convergent input from an apparently random subset of glomeruli, and aO1 odor responses are anatomically discontinuous and distributed (Stettler and Axel, 2009). This random connectivity profile has computational advantages for specifically learning arbitrary stimulus feature combinations (Ganguli and Sompolinsky, 2012). Principal cells in aO1 are recurrently connected and the strength of odor-evoked responses in these cells is largely determined by recurrent rather than bulbar inputs (Franks et al., 2011; Poo and Isaacson, 2011).

**Box 1. The many primary cortical areas of the olfactory system.**

Multiple cortical structures receive direct bulbar inputs and may thereby be considered part of the primary olfactory cortex. These include anterior olfactory cortex [AOC, often referred to as anterior olfactory nucleus despite having a cortical architecture (Haberly, 2001; McGinley and Westbrook, 2011)], piriform cortex [PC; including anterior (aPC) and posterior (pPC) subdivisions], and lateral entorhinal cortex (LEC). We use the convention that the primary olfactory cortex (O1) includes all cortical areas with direct bulbar inputs. There are anatomical and functional differences between these areas that suggest separate aspects of odor processing are distributed across O1 subsections, and these follow a general anteroposterior gradient. Afferent projections from the OB are denser to the aO1 compared to the pO1 (Wang et al., 2020). Connectivity profiles between O1 areas suggests that there are more feed-forward projections going from anterior to posterior than feed-back projections from posterior to anterior (Yang and Sun, 2015). The long axis of the piriform cortex is characterized by detailed odor encoding anteriorly and coarser category encoding posteriorly (Gottfried et al., 2006). This representational gradient bears similarities with the long axis of the hippocampus, where fine spatial detail is represented dorsally and broader spatial context ventrally (Strange et al., 2014). aPC is densely connected with the orbitofrontal cortex and is suggested to encode behaviorally relevant (action-based) associations with odor input (Haberly, 2001). aPC neural responses display pattern completion and pattern separation and are dynamically shaped by shifting perceptual demands (Shakhawat et al., 2014). There is little data on whether other parts of O1 display these characteristics. Compared to aPC, odor responses in pPC are more strongly modulated by odor-outcome associations which likely result from its more prevalent connections with basolateral amygdala. The pPC is therefore a prime location for integrating emotional associations with odors (Majak et al., 2004; Calu et al., 2007). While most O1 areas receive inputs from OB mitral cells, the most anterior section (AOC) receives exclusive input from a distinct OB projection neuron, the tufted cell (Igarashi et al., 2012). AOC is also proposed to be the site of initial odor identity encoding (Haberly, 2001). Odor identity encoding must be independent of AOC to a certain extent, however, as temporary AOC lesions leave odor discrimination performance unaffected (Levinson et al., 2020). Rats in this study were already familiarized with the odor stimuli, leaving open the possibility that AOC is important for odor identity encoding during learning but not subsequently. The primary olfactory cortex is the only cortical region that receives strong primary sensory inputs in combination with substantial direct input from the hippocampus proper, and this unique input combination is most prominent in the AOC (Aqrabawi and Kim, 2018a,b). Representations in AOC reflect context, and an intact AOC is required for odor-context learning (Aqrabawi and Kim, 2020; Levinson et al., 2020). Through this combined input, spatial context and/or episodic memory is therefore likely integrated as part of the initial cortical representation of odor identity. Hippocampal inputs may also play a part in contextually cued pattern completion and pattern separation in primary olfactory areas and perhaps bias categorical learning toward or away from *de novo* odor representations depending on the situation. At the posterior end of O1 lies LEC, one of the main cortical inputs to the hippocampus. Information from the olfactory system therefore has an unusually direct route into the hippocampus. Although the O1 includes many distinct structures, in this perspective our focus is on the aO1 where characteristics of categorical learning have been best documented.

### aO1 Odor Representations Are Synthetic

In the rat OB, exposure to a mixture of two monomolecular odor components causes neural cross-habituation to the isolated mixture components in subsequent exposures (Wilson, 2003). OB responses to mixtures therefore likely involve the same neural ensembles that activate in response to the isolated components. While this cross-habituation is also observed in aO1 in response to short (10 s) mixture exposures, longer (50 s) mixture exposures eliminates this effect (Wilson, 2003). This suggests that odor mixture familiarization on the order of minutes is sufficient to recruit distinct neural ensembles to encode the mixture as separate from the identity of its components. Odor encoding in aO1 therefore depends on experience and is synthetic because the collection of features is represented differently from the features themselves. aO1 odor mixture representations predict perceptual performance, implying that they form the basis of odor perception (Wilson et al., 2020).

### aO1 Odor Representations Are Categorical and Invariant

Responses to familiarized odor mixtures remain stable even if single mixture components are omitted, a process termed pattern completion (Barnes et al., 2008). Pattern completion of odor responses in aO1 critically depends on recurrent aO1 circuitry, suggesting that categorical learning occurs already within aO1 (Bolding et al., 2020). Furthermore, learning to perceptually discriminate between very similar odor mixtures causes neural activation patterns in aO1 to diverge despite no change to the stimuli, known as pattern separation (Shakhawat et al., 2014). The timescale of neural pattern separation developing in aO1 ensembles matches that of behavioral effects of discrimination training (Chapuis and Wilson, 2011; Shakhawat et al., 2014). These encoding characteristics highlight that odor representations in aO1 reflect perceptual classification rather than exact chemical details present in the input and are flexible according to perceptual demands.

### Natural Odors Are Mixtures and Interpretable Through Association

The rather unusual primary cortical attributes of aO1 make sense considering that natural odors are not monomolecular, and behavioral interpretation of natural odors is not usually defined by their molecular structure *per se*. Instead, most natural odors are mixtures of molecules that become useful for adaptive behaviors through association. While individual odor molecules may belong to several distinct phenomena, it is the joint probability distribution of molecules across exposures that qualifies unique odor representations. But how do these representations form? Because of the high dimensionality and discrete nature of the olfactory input space, and the synthetic format of aO1 representations, it is experimentally straightforward to design unique odor stimuli for aO1. Using novel odor stimuli offers a way to carefully study the formation and characteristics of categorical representations within aO1.

## Statistical Learning of Categorical Representations

Categorical learning is a statistical and dynamic process that depends on experience and perceptual demands. It is thought that categorical representations emerge with experience through associative learning that extracts and binds together invariant feature axes from distinct stimulus configurations. To become separable in neural activity space, these feature axes should minimize overlap between distinct representations. Categorical sensory configurations can this way be thought of as distributions living in a high-dimensional feature space, and determining the combination of feature axes that best define and separate various distributions represents a statistical learning problem (Turk-Browne et al., 2009). Because classification of sensory configurations can depend on behavioral relevance, context and novelty, the underlying structure of categorical sensory representations must be flexible to accommodate dynamic perceptual demands imposed by the environment. How neural circuits achieve this feat is ill understood.

**FIGURE 1:**
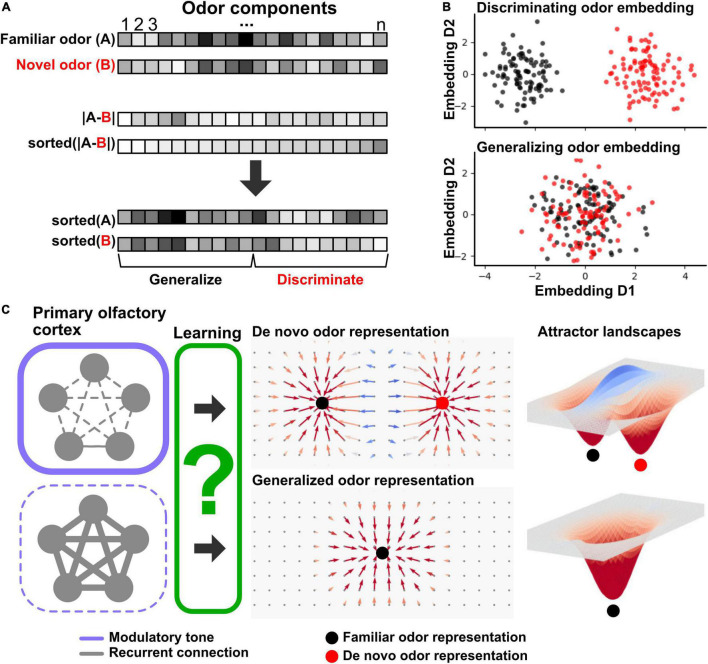
Schematic of categorical learning in primary olfactory cortex. **(A)** Odor stimuli as high-dimensional feature vectors. (Top) a familiar odor (A) is characterized by a distribution of odor component concentrations (horizontal box row, high concentration indicated by dark coloring). A novel odor (B) is shown below that has considerable overlap with odor A in its component distribution. (Middle) absolute difference in component concentration between odors A and B (top box row) and the same difference but sorted according to value (bottom box row). (Bottom) the original odors A and B component distributions shown in panel **(A)** but sorted according to component similarity. The left-hand side reflects components that are most similar between the odors, while the right-hand side reflects components that are the most dissimilar, supporting generalization and discrimination respectively. **(B)** Low dimensional embeddings of odors A and B according to maximum discrimination (top) or generalization (bottom). Each dot represents one exposure to an odor (odors A and B, color-coded by black and red, respectively). The embeddings are computed using the top two (top panel) and bottom two (bottom panel) principal components (D1 and D2) of component distributions across the odors. The embeddings highlight that within overlapping odor distributions, there are embeddings that may dynamically cater to either discrimination (top) or generalization (bottom) depending on component weighting. **(C)** Schematic of proposed role of modulatory system regulation of generalization-discrimination balance during odor learning through regulation of recurrent primary olfactory cortical connections. (Left) Recurrent connections (gray lines) between neurons (gray circles) represent primary olfactory cortical ensembles. When faced with a novel odor stimulus, modulatory tone (purple frames) regulates activity in recurrent connections which biases learning between generalization and discrimination. High modulatory tone inhibits recurrent activity (top, indicated by thick frame and thin connections), while low modulatory tone boosts recurrent activity (bottom, indicated by thin frame and thick connections). Through learning (green box, unknown mechanisms), the primary olfactory cortex either sets up categorical representations that discriminate a novel odor into a *de novo* representation separate from a familiar odor (top panel) or generalizes the novel odor into an existing familiar odor representation (bottom). The middle panels schematically illustrate neural phase space diagrams. The 2D surface represents variable neural ensemble activations that then through attractor dynamics converge to an invariant and categorical response (vectors leading to filled circles). The top panel shows the neural phase space resulting from a *de novo* categorical representation reflected in two fixed point attractors (black and red circles, familiar and *de novo* representations, respectively), while the bottom panel shows the neural phase space resulting from generalizing a novel odor into a familiar representation as reflected in a single fixed-point attractor (black circle). (Right) attractor dynamical landscapes associated with the neural phase space diagrams in the middle. Filled circles denote attractor basins.

Because the mixture component distribution of natural odor stimuli typically varies across experiences, the aO1 faces a predicament when encountering a novel stimulus, especially if the novel odor has considerable component overlap with familiar odors. Should the novel stimulus be integrated as a new example of a familiar odor representation, or as a *de novo* odor representation? To incorporate a novel stimulus into an existing representation, the statistics of this representation must be changed to *include* the new mixture distribution. A functional requirement for generating *de novo* stimulus representations is to *separate* the novel stimulus representation from existing representations. Pattern completion in aO1, which reflects stimulus generalization, depends on recurrent circuits that implement attractor dynamics, and experimentally inhibiting recurrent connectivity in aO1 changes several features of the attractor landscape (Bolding et al., 2020). This points to recurrent aO1 connections as a target for dynamically controlling the *balance* between representational generalization and discrimination in odor learning. A central question is how the brain dynamically controls this balance. As described in the next section, the concerted activity of several neuromodulatory systems is a good candidate mechanism for how dynamic control over both attractor-like activity and synaptic learning rules within the aO1 recurrent circuitry is implemented (see Figure 1).

## Controlling Categorical Learning in Olfactory Cortex

While there is a plethora of neuromodulators that might participate in regulating aO1 dynamics, here we will discuss a subset that is particularly implicated through the existing literature. The neuromodulator acetylcholine (ACh) affects both behavioral and neural measures of odor discrimination and generalization. ACh selectively inhibits intrinsic activity in recurrently connected cortices without affecting afferent input responses (Hasselmo and Bower, 1992), proposed to be a mechanism that switches between recognizing familiar stimuli to rapidly encoding novel stimuli in auto-associative networks (Hasselmo et al., 1992). Interestingly, the same mechanism also minimizes interference between responses to novel and familiar inputs (De Rosa and Hasselmo, 2000). Pharmacological manipulation studies have shown that ACh plays a critical role in perceptual learning: odor discrimination learning is impaired by muscarinic ACh receptor antagonists and facilitated by agonists (Chapuis and Wilson, 2013). Importantly, these effects are limited to the acquisition phase, pointing to a central role for ACh in the *initial* formation of cortical odor representations.

Noradrenaline (NA) also affects odor learning and is involved in novelty processing. Like ACh, NA activation selectively suppressed aO1 recurrent activity while leaving afferent inputs unaltered (Hasselmo et al., 1997), and was proposed to function as a reset signal in response to novelty, shifting the system from retrieval mode to encoding mode (Bouret and Sara, 2005; Grella et al., 2021). Odor discrimination learning is impaired by phasic stimulation of the noradrenergic locus coeruleus (LC) and facilitated by NA antagonists (Ghosh et al., 2021). In the hippocampus (another recurrently connected cortical structure), LC stimulation can induce ensemble reconfigurations (remapping) in a familiar environment, suggesting that it is sufficient to initiate formation of novel representations even in the absence of novelty. In a novel environment, inhibition of LC causes reinstatement of an existing hippocampal ensemble, demonstrating that LC is necessary for generating novel representations (Grella et al., 2019) at least in the hippocampal system. LC is a source of dopamine (DA) in addition to NA. DA is involved in rapid generation of novel cortical representations. While novel combinations of familiar stimuli lead to DA release from the ventral tegmental area, novel stimulus features cause DA release from the LC, which is required for the formation of episodic representations in the hippocampus (Duszkiewicz et al., 2019). In the olfactory system, NA is necessary for discrimination learning and pattern separation, and both NA and DA antagonists block the effects of phasic LC stimulation on olfactory discrimination learning (Shakhawat et al., 2015; Ghosh et al., 2021). An interesting extension from these findings is that attentional state may through neuromodulators change categorical learning rules (Thiele and Bellgrove, 2018).

## Catastrophic Forgetting and Odor Learning

### Natural Odors Are Extraordinarily Variable but Specific Spatiotemporal Contexts Tend to Associate With Distinct Odor Subsets

As a consequence of natural exploration, it is highly likely that an animal in the wild continually encounters and learns novel odor stimuli throughout its lifetime. With continual learning, the olfactory system must maintain the established representational catalog while incorporating *de novo* categorical representations. Abrupt destructive interference between new learning and previously learned representations is termed catastrophic forgetting [as opposed to advantageous forgetting (Migues et al., 2016)] and is a challenge in state-of-the-art artificial neural networks (ANNs) (Kirkpatrick et al., 2017; Kaplanis et al., 2018). The brain clearly overcomes this problem; the rodent olfactory system has a large capacity to learn novel inputs with minimal interference, even when previously learned representations are not frequented (Staubli et al., 1987). ANNs learn by modifying connection weights between model neurons, so learned representations are therefore sensitive to changes in connection weights. Continual ANN learning involves modifying these weights in response to training sets that contain novel stimuli without revisiting familiar stimuli. Weight changes introduced by the novel stimuli can cause abrupt forgetting of previously learned representations because it is impossible for the network to simultaneously update weights required to implement the new representation while preserving weights associated with existing representations. Categorical learning in aO1 may provide a better understanding of the differences between these learning rules in biological versus artificial neural networks.

Arguably, because odor representations are synthetic and categorical, generating *de novo* odor representations requires plasticity at distributed recurrent aO1 synapses. As it is connectional plasticity that causes catastrophic forgetting in ANNs, it implies that any biological mechanism that implements continual categorical learning also needs to modulate learning rules at the relevant synapses to avoid catastrophic forgetting. In Kirkpatrick et al. (2017), part of the proposed solution was to make synaptic learning rates inversely proportional to their influence in previously learned representations. This in effect forces learning new stimuli into separate subspaces of the network that preserves robustness in the remaining space. In subsequent work (Kaplanis et al., 2018), more complex and biologically inspired connections that implement synaptic variables on multiple timescales offered improved robustness against catastrophic forgetting. Both solutions may have analogies to structural plasticity at dendritic spines as observed in cortical networks.

### Neuromodulators and Representational Robustness in aO1 During Continual Learning

Apart from scaling recurrent excitability, neuromodulators are also perfectly positioned to control several parameters of synaptic plasticity in aO1. A large body of literature, although mostly based on *ex vivo* and *in vitro* experiments, has demonstrated that several modulatory systems powerfully regulates metaplasticity—the plasticity of plasticity—across a range of physiologically relevant timescales (see Brzosko et al., 2019 for review). Specific to our discussion, modulation can drastically alter local learning rules that may be key to how continual categorical learning is implemented in aO1 without interfering with previously learned representations. For instance, modulatory inputs to the hippocampus can flip the polarity of long-term plasticity (Sugisaki et al., 2016) and set the “contrast” of plasticity by changing the threshold for postsynaptic spiking needed to induce plasticity in prefrontal cortex (Couey et al., 2007). Depending on the combinatorial activity of several neuromodulators influenced by behavioral state, synaptic dynamics in aO1 may enter specific regimes that cater to distinct learning criteria including protection of learned representations from formation of new representations. However, no experiments to our knowledge have so far targeted these interactions *in vivo* in recurrent networks like aO1.

## Novelty Detection in the Primary Olfactory Cortex

A central question in categorical learning is what defines a novel stimulus? Although one can generate novel stimuli in any sensory modality, there is a difference between a novel combination of familiar features and novel features in themselves. In the visual system for example, it is not the features in V1 that are novel, rather the constellation of these in higher order cortices. In the olfactory system, single glomerular activations represent familiar features, and aO1 representations their unique constellation. We have little insight into how odor novelty is detected in the mammalian olfactory system. One structure that is intimately involved with novelty detection is the hippocampus. Part of the aO1 (AOC) receives a particularly strong direct projection from the hippocampus that might take part in novelty detection. However, the speed with which rats can detect and respond to odor novelty is faster than a single sniff (50–100 ms) (Wesson et al., 2008). Inactivation of direct hippocampal inputs to aO1 does not impair novel odor detection in mice (Aqrabawi and Kim, 2018a). It is therefore likely that odor novelty detection is computed very rapidly and locally within the olfactory system itself. Future work is needed to better our understanding of the mechanisms that implement novelty detection in the olfactory system. However, the *threshold* for novelty detection may be regulated through modulation of local recurrent activity and plasticity in aO1.

## Discussion

Rodent olfaction provides an excellent model system for understanding how the brain flexibly generates novel categorical representations and how these are integrated with existing representations. The aO1 is a unimodal sensory area positioned only two synapses from the sensory organ, minimizing synaptic non-linearities that otherwise obscure transformations from sensory inputs to cortical responses. Unlike in the hippocampus, where novelty detection unfolds with exploration and is therefore more difficult to determine with temporal precision, novel odor stimuli and their detection by the experimental subject can be precisely timed. Novel odor stimuli with predefined feature correlations can readily be designed using odor mixtures, making it trivial to repeatedly provide novel stimuli to the same subject. It may therefore be possible to disentangle activity motifs on the population level that are specific to novelty detection and continual categorical learning versus the stimuli themselves.

Several outstanding questions about categorical learning in aO1 remain.

(1) Do familiar odor representations in aO1 undergo reformatting in response to *de novo* odor representations, and if so, does that reformatting reflect statistical learning rules that dynamically optimizes separability between representations?
(2) How does neuromodulatory activity change population dynamics on short (seconds) and intermediate (minutes-hours) timescales within aO1?
(3) Can catastrophic forgetting of established odor representations be induced by manipulating modulatory activity in aO1 during continual learning?
(4) How does aO1 activity develop across sniffs during first exposure to a novel odor?
(5) What distinguishes subsections of aO1 activity during categorical learning; is acquisition and storage of representations implemented by separate neural populations?

Making progress will require recording large ensembles of aO1 neurons that are trackable across recording sessions. Furthermore, understanding the dynamics of population activity on the timescale of individual sniffs requires that the recording technique offers high temporal resolution, and that odor delivery is controlled with high temporal precision. Recent advances in electrophysiological recording techniques, such as Neuropixels silicon probes (Steinmetz et al., 2021) that allow simultaneously recording on the order of 10^3^ neurons (Gardner et al., 2022), offer attractive methodological approaches that meet these requirements. Virtual reality techniques are well established in systems neuroscience (Fiser et al., 2016) and a good match with electrophysiology given the parametric stimulus control and richness of behavioral readout that it supports. Finally, optogenetic or chemogenetic techniques can be leveraged to gain experimental control over specific modulatory system inputs to aO1.

In conclusion, categorical learning represents a fundamental neural computation that is attractive on several levels across several academic disciplines, and the olfactory system offers unique experimental advantages toward understanding this neural computation.

## Data Availability Statement

The original contributions presented in this study are included in the article/supplementary material, further inquiries can be directed to the corresponding authors.

## Author Contributions

TS and HS conceptualized and wrote the manuscript. Both authors contributed to the article and approved the submitted version.

## Conflict of Interest

The authors declare that the research was conducted in the absence of any commercial or financial relationships that could be construed as a potential conflict of interest.

## Publisher’s Note

All claims expressed in this article are solely those of the authors and do not necessarily represent those of their affiliated organizations, or those of the publisher, the editors and the reviewers. Any product that may be evaluated in this article, or claim that may be made by its manufacturer, is not guaranteed or endorsed by the publisher.
